# Association between TERT promoter polymorphisms and acute myeloid leukemia risk and prognosis

**DOI:** 10.18632/oncotarget.4668

**Published:** 2015-07-23

**Authors:** Mohamed Ali Mosrati, Kerstin Willander, Ingrid Jakobsen Falk, Monica Hermanson, Martin Höglund, Dick Stockelberg, Yuan Wei, Kourosh Lotfi, Peter Söderkvist

**Affiliations:** ^1^ Department of Clinical and Experimental Medicine, Linköping University, Linköping, Sweden; ^2^ Department of Haematology and Department of Clinical and Experimental Medicine, Linköping University, Linköping, Sweden; ^3^ Department of Medical and Health Sciences, Linköping University, Linköping, Sweden; ^4^ Department of Immunology, Genetics and Pathology, Rudbeck Laboratory, Uppsala University, Uppsala, Sweden; ^5^ Division of Hematology, Department of Medical Sciences, Uppsala University, Uppsala, Sweden; ^6^ Section for Hematology and Coagulation, Department of Internal Medicine, Sahlgrenska University Hospital, Gothenburg, Sweden; ^7^ Department of Hematology, County Council of Östergötland, Linköping, Sweden

**Keywords:** TERT, SNV, AML, prognostic markers

## Abstract

Telomerase reverse transcriptase gene (*TERT*) promoter mutations are identified in many malignancies but not in hematological malignancies. Here we analyzed *TERT* and protection of telomeres 1 gene (*POT1)* mutations, and four different *TERT* SNVs in 226 acute myeloid leukemia (AML) patients and 806 healthy individuals in a case referent design, where also overall survival was assessed. A significant association for increased risk of AML was found for *TERT* SNVs, rs2853669 (OR = 2.45, *p* = 0.00015) and rs2736100 (OR = 1.5, *p* = 0.03). The overall survival for patients with CC genotype of rs2853669 was significantly shorter compared to those with TT or TC genotypes (*p* = 0.036 and 0.029 respectively). The influence of TERT rs2853669 CC on survival was confirmed in multivariable Cox regression analysis as an independent risk biomarker in addition to high risk group, higher age and treatment. No hot spot TERT promoter mutations at −228C > T or −250C > T or *POT1* mutations could be identified in this AML cohort. We show that rs2853669 CC may be a risk factor for the development of AML that may also be used as a prognostic marker to identify high risk normal karyotype -AML (NK-AML) patients, for treatment guidance.

## INTRODUCTION

It is estimated that more than 90% of all cancers have increased telomerase activity [1] which correlates with immortalization, resistance to senescence and apoptosis, and telomere elongation. TERT is the transcriptional catalytic subunit for telomerase activity [2] and is considered to have a critical role in tumor formation. Therefore, genetic variants and somatic alterations in the *TERT* gene may affect telomerase function and contribute to the development of cancer as well as the outcome of chemotherapy. Up regulation of TERT expression is abundantly reported in somatic cells and two hot spot mutations in the *TERT* promoter, −228C > T and −250C > T, were recently reported in several different solid tumors *e.g* melanoma and gliomas [3–6]. These mutations have strong clinical implications with worse prognosis and poor survival and may represent a novel therapeutic target [6]. Hematological malignancies are not reported to be subject for somatic promoter mutations in the *TERT* gene but display enhanced telomerase activity and shortened telomeres [7]. An alternative or additional mechanism to altered telomere length regulation, in the absence of *TERT* mutations, may be the presence of single nucleotide variants (SNVs) in the *TERT* gene influencing activity and/or expression. Meta-analysis of 85 studies of SNV's and cancer types, but not hematological malignancies revealed associations for several *TERT* SNV's [8]. Telomere integrity is also regulated by the protection of telomeres 1 (*POT1*) gene and the POT protein is responsible for recruiting telomerase to the single-stranded 3′ telomere repeats and consecutively limiting telomere elongation by telomerase [9]. Andrew et al. identified twelve (3.5%) *POT1* somatic mutations in chronic lymphocytic leukemia (CLL), the majority affecting the two oligonucleotide/oligosaccharide-binding (OB) folds suggested to have an essential role in binding to the telomeric repeats and for protein function [10]. *POT1* loss-of-function mutations are prevalent in familial melanoma [11].

TERT also possess telomere independent functions in tumor formation and other human diseases, regulating Wnt-dependent transcription [12], mitochondrial function and apoptosis [13] and DNA damage response [14]. Recently, it was shown that *TERT* interact with NFκB and co-activate the expression of several genes, including cytokines, such as IL-6 and TNFα, that are critical for inflammatory reactions and cancer progression [15, 16].

Genetic alterations resulting in enhanced telomerase activity have recently been implicated in a variety of bone marrow failure syndromes such as acute myeloid leukemia [7, 17–18] inducing an expansion of undifferentiated myeloid hematopoietic stem cell progenitors, but so far no somatic mutations in either *TERT* or *POT1* genes have been described in acute myeloid leukemia (AML).

AML is a genetically heterogeneous disease with various cytogenetic abnormalities affecting clinical outcome. AML with cytogenetically normal karyotype (NK-AML) have varying outcomes and there is a lack of risk markers to identify patients with worse prognosis. Mutations in the nucleophosmin 1 (*NPM1*), fms-related tyrosine kinase 3 (*FLT3*) and CCAAT/enhancer binding protein alpha (*CEBPA*) genes, are clinically important prognostic markers of outcome and survival particularly for NK-AML [19, 20].

Mutations in *NPM1* indicates a favorable factor for achieving complete remission (CR) [21]. In contrast, *FLT3* internal tandem duplication (ITD) is present in 30% of NK-AML patients, and associated with increased relapse rates, and decreased overall survival among NK-AML patients [22–25]. The CEBPA is the founding member of a family of related leucine zipper transcription factors that play important roles in myeloid differentiation [26]. Mutations in *CEBPA^double-mut^* are seen in 5% to 14% of AML and have been associated with a favorable clinical outcome. Only double mut is considred. Still a large proportion of NK-AML patients with intermediate risk are lacking prognostic markers to guide in treatment decisions.

Several studies investigating the impact of individual SNVs on prognosis and survival in AML have been suggested, but so far not reached the clinic e.g XPA and XPD variants, ABCB1, WT1 and IDH variants [27–31]. Most of them are based on candidate pathway approaches suggesting some significant SNV(s) to reflect the prognosis of the patients. We selected four *TERT* SNVs on the basis of previous reports of their association with the risk for several other forms and hallmarks of cancer and its potential as a biomarker of clinical outcome in AML.

## RESULTS

### *TERT* mutation and polymorphisms genotyping analysis

Mutation analysis of the *TERT* promoter and selected *POT1* exons disclosed no mutations in our AML patient cohort. *TERT* 1062A> T (rs35719940) that has been identified as a susceptibility mutation in an Egyptian AML population, was found in 10/249 patients (4%) and in 22 of 806 healthy control (2.7%), (OR = 1.47, 95% CI 0.68–3.14, *p* = 0.21). Nevertheless rs35719940 showed no association with an increased risk for AML or effect on survival.

**Figure 1:**
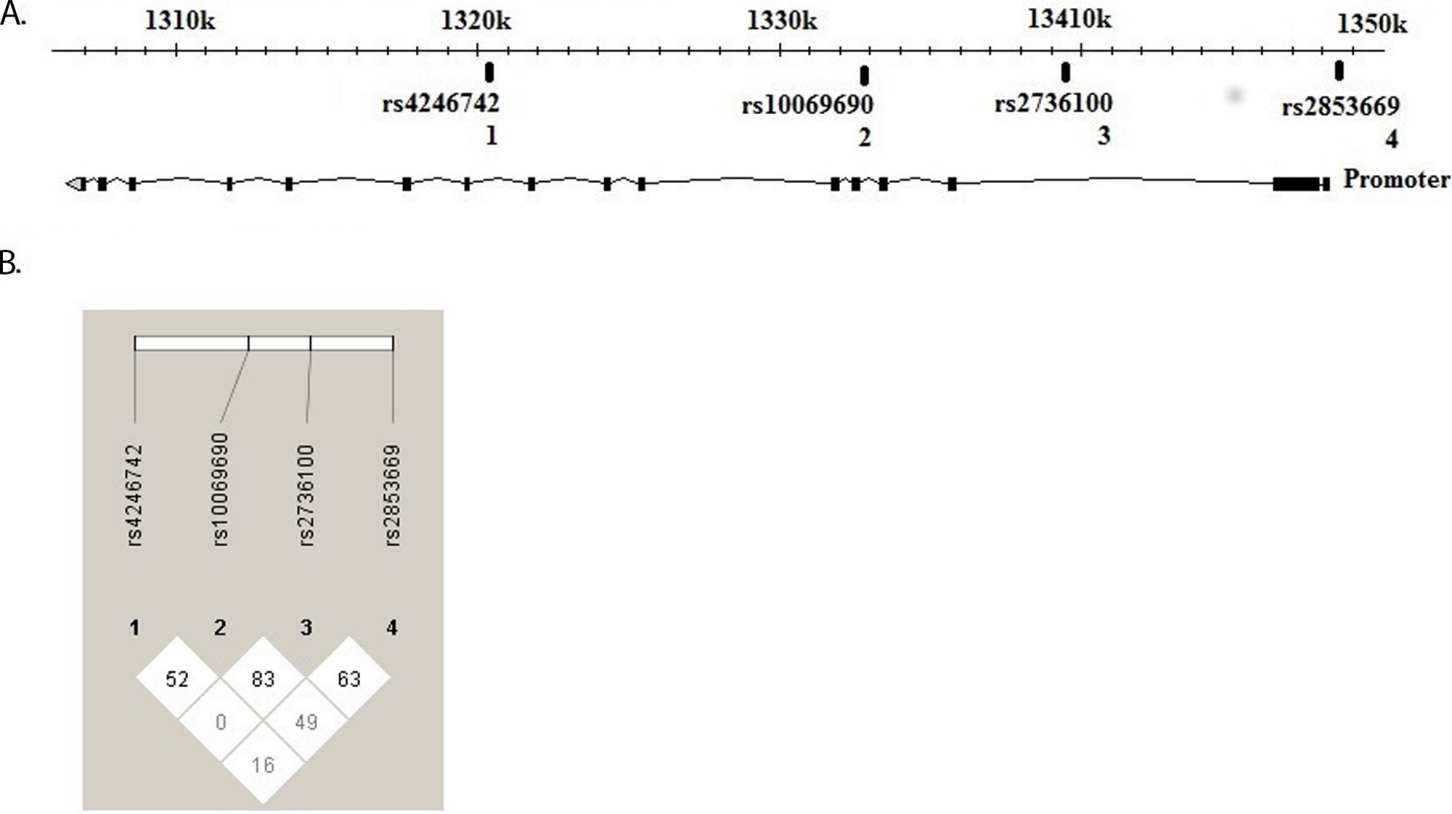
*TERT* gene, SNVs localization and disequilibrium Localization of *TERT* SNVs, SNV1 (rs4246742), SNV2 (rs10069690), SNV3 (rs2736100) located at introns 8, 4 and 2 respectively and SNV 4 (rs2853669) located at the promoter region. (HapMap Data Rel 28/phase II+III, October 2010, on NCBI B36 assembly, dbSNP b126) **A.** Linkage disequilibrium showed a modest linkage between rs2853669 and rs2736100. (HaploView version 4.2) **B.**

Genotyping of the four SNVs in the *TERT* gene in this study, revealed an increased risk for AML of the rs2853669 CC genotype (OR = 2.45, 95% CI 1.54–3.88, *p* = 0.00015) (Table 1). Homozygosity for the minor allele of rs2736100 (CC) disclosed a modest but still significantly increased risk for AML (OR = 1.5, 95% CI 0.98–2.29, *p* = 0.03) (Table 1). None of the other analyzed *TERT* SNV's showed any overall association with risk for AML. rs2853669 (C > T) is located in the *TERT* promoter at −246 upstream the ATG start codon, in proximity to the somatic mutation hot spots identified in several solid cancers. rs2736100 (C > A), rs10069690 (C > T) and rs4246742 (C > T) are located in introns 2, 4 and 8 respectively of the *TERT* gene (Figure 1A) and linkage disequilibrium (LD) analysis for the different SNV's showed a modest linkage between rs2853669 and rs2736100 (Figure 1B).

**Table 1:** Genotype distribution of the different polymorphisms in AML patients and normal control population investigated in the study and their association with AML susceptibility

Polymorphisms	AML n (%)	Controls *n* (%)	OR (95% CI)	*p*-Value
rs2853669				
TT	89 (39.38)	373 (47.88)		
TC	99 (43.8)	341 (43.77)	1.21 (0.88 – 1.67)	0.13
CC	38 (16.81)	65(8.34)	2.45 (1.54 – 3.88)	**0.00015**
TC + CC	137	406	1.41 (1.04 – 1.91)	0.01
Total	226	779		
rs10069690				
CC	118 (52.21)	409 (53.32)		
CT	93 (41.15)	319 (41.60)	1.01 (0.74 – 1.37)	0.5
TT	15 (6.63)	39 (4.68)	1.33 (0.71 – 2.5)	0.22
CT + TT	108	358	1.04 (0.77 – 1.4)	0.41
Total	226	767		
rs2736100				
AA	48 (21.23)	201 (25.5)		
AC	113 (50)	406 (51.53)	1.16 (0.79 – 1.7)	0.24
CC	65 (28.76)	181 (22.97)	1.5 (0.98 – 2.29)	**0.03**
AC + CC	178	587	1.26 (0.88 – 1.81)	0.1
Total	226	788		
rs4246742				
TT	156 (69.02)	520 (66.41)		
TA	65 (28.76)	240 (30.66)	0.9 (0.65 – 1.25)	0.29
AA	05 (2.21)	23 (2.93)	0.72 (0.27 – 1.93)	0.35
TA + AA	70	263	0.88 (0.64 – 1.22)	0.25
Total	226	783		

### Impact of *TERT* rs2853669 on overall survival

**Figure 2:**
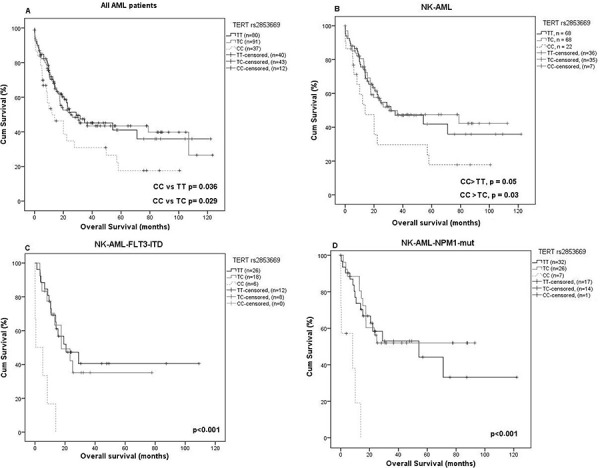
Differences in overall survival (OS) depending on *TERT* rs2853669 genotypes In entire group and in NK-AML group homozygous CC genotype was significantly associated with a shorter OS **A.** and **B.** In *FLT3*-ITD-positive patients, homozygous CC genotype was significantly associated with a shorter OS **C.** the mean OS was 4.6 vs. 11.8 and 12.6 months for CC vs. TT and TC genotype, respectively, *p* < 0.001, **D.** The same result was found for *NPM1* mutated patients, the mean OS was 5.4 vs. 18.4 and 13.8 months CC vs. TT and TC respectively *p* < 0.001.

The rs2853669 CC genotype is firmly associated with an increased risk for AML (*p* = 0.00015) and Kaplan Meier survival analysis revealed a decrease in overall survival (OS). A significant difference in OS was evident for all AML patients as well as NK-AML patients, being homozygous CC for rs2853669 compared to TT or TC heterozygotes (*p* = 0.036 and 0.029, respectively) for all AML and *p* = 0.05 and 0.03, respectively, for NK-AML patients, (Figure 2A and 2B). Stratification of NK-AML patients according to *FLT3* or *NPM1* status, revealed a significantly reduced OS for NK-AML rs2853669 CC genotypes among *FLT3*-ITD or *NPM1* non-mutated patients (4.6 months compared to 11.8 or 12.6 months ( *p* < 0.001) for the TC or TT genotypes, respectively (Figure 2C). A similar pattern was observed when NK-AML patients were stratified according *NPM1* status. *NPM1*-mutated patients with a CC genotype showed a significantly shorter OS compared to TC or TT (5.4 vs 13.84 and 18.4 months, *p* < 0.001) (Figure 2D). However rs2736100 that showed a modest risk for AML, had no effect on survival in our AML cohort.

To evaluate whether *TERT* rs2853669 is an independent prognostic marker for survival in the entire AML group (Table 2) and when stratified according to karyotype (Table 3 and 4), multivariable Cox-regression analysis was performed with covariates including age, risk group and treatment (chemo only or chemo + allogeneic stem cell transplantation) (Table 2). The rs2853669 CC genotype was identified as an independent predictor for patient survival, in entire cohort and in NK-AML patients ( *p* = 0.024 and 0.022, respectively), in addition to older age ( *p* = 0.001 and 0.003, respectively) and classification in a high risk group ( *p* = 0.002 and 0.061, respectively). The Cox-regression analysis also showed that transplantation is an independent predictor for increased survival (*p* = 0.002) (Table 2).

**Table 2:** Cox regression of overall survival in entire AML cohort

*Covariates*	HR	95% CI	*p*-Value
Age	1.03	1.01 – 1.05	**0.001**
Risk group			
• *Intermediate risk^a^	1.76	0.92 – 3.37	**0.038**
• *High risk^a^	3.23	1.51 – 6.91	**0.002**
TERT rs2853669 C/T^b^	1.35	0.87 – 2.10	0.110
TERT rs2853669 C/C^b^	1.74	1.03 – 2.94	**0.024**
Treatment^c^	0.5	0.29 – 0.84	**0.002**

a Compared to low risk,

b Compared to T/T

c Chemo + allo-SCT compared to chemo only

*Intermediate risk: FLT3-ITD positive/NPM1 mutation positive or FLT3-ITD negative/NPM1 mutation negative or FLT3-ITD negative/monoallelic and or CEBPA wild type patients, *High risk: FLT3-ITD positive/NPM1 mutation negative NK-AML patients, *Low risk: FLT3-ITD negative/NPM1 mutation positive or FLT3-ITD negative/biallelic CEBPA mutation patients.

**Table 3:** Cox regression of overall survival in NK-AML patients

*Covariates*	HR	95% CI	*p*-Value
Age	1.03	1.01 – 1.06	**0.003**
Risk group			
• *NK-intermediate risk^a^	1.49	0.77 – 2.87	0.22
• *NK-high risk^a^	2.32	0.94 – 5.67	0.061
TERT rs2853669 C/T^b^	1.22	0.75 – 2	0.40
TERT rs2853669 C/C^b^	2.08	1.11 – 3.88	**0.022**
Treatment^c^	0.56	0.3 – 1.02	0.061

a Compared to low risk

b Compared to T/T

c Chemo + allo-SCT compared to chemo only

**Table 4:** Cox regression of overall survival in aberrant AML karyotype

*Covariates*	HR	95% CI	*p*-Value
Age	1.04	1.001 – 1.086	**0.046**
Risk group			
• *Aberrant K-intermediate risk^a^	1.49	0.77 – 2.87	0.235
• *Aberrant K-high risk^a^	2.46	0.92 – 6.58	0.073
TERT rs2853669 C/T^b^	1.22	0.75 – 2	0.409
TERT rs2853669 C/C^b^	0.8	0.22 – 2.89	0.736
Treatment^c^	0.34	0.1 – 1.16	0.086

a Compared to low risk

b Compared to T/T

c Chemo + allo-SCT compared to chemo only

### Correlation between *TERT* expression and other pro-inflammatory cytokines

Since the TERT protein is proposed to co-activate NFκB to enhance gene-expression of *e.g* IL-6, TNFα and other pro-inflammatory cytokines, contributing to tumor progression, we analyzed the relative mRNA levels on thirty-six AML patients with different rs2853669 genotypes (Supplementary Figure S1A–S1C). The CC genotype increased the TERT mRNA levels 2.17 fold, a non-significant increase. On the other hand, both IL-6 and TNFα were significantly increased, 6.25 and 3.58 fold respectively, for the CC genotypes compared to CT and TT genotypes ( *p* < 0.04 and 0.05, respectively, Supplementary Figure S1B and S1C).

## DISCUSSION

In the promoter region, close to the melanoma and glioblastoma hot spot *TERT* mutations, there is a SNV rs2853669 (−245 T > C), which minor allele destroy a binding site for the Ets2 transcription factor, with an allele frequency in Europeans of 29% (1000 Genome project, http://www.Ensembl.org) and in the Swedish population of 28.6% (Table 1). We analyzed this and three additional intragenic SNVs for association to the risk of developing AML, in relation to clinical prognostic and predictive biomarkers and to overall survival in AML (*n* = 226). We found that the CC genotype of the *TERT* promoter SNV (rs2853669) and also the intronic SNV rs2736100 were associated with increased risk of AML. Previous studies have recognized this SNV as having an important role in other cancers, like lung and breast cancers [32, 33] and in non-small cell lung cancer (NSCLC) tissues [16].

The rs2736100 is located in intron 2 of *TERT* within a putative regulatory region [8, 34] and is the most studied SNV of the *TERT* gene. It has been described in 46 studies enrolling in total 74785 case subjects with 11 solid tumor types and 115726 control subjects [8]. However, no blood tumors were included in this meta-analysis and our study now add AML to the list of tumors with a moderate influence on AML risk associated to the rs2736100 SNV. Sheng and colleagues showed that rs2736100 CC genotype is associated with lower telomerase activity and longer telomere length, compared with the wild type allele, but not affecting *TERT* mRNA expression [35]. Otherwise, rs2736100 is close to mutations known to alter telomerase activity [8]. Rachakonda and Hsu showed that rs2853669 CC genotype influence telomerase activity and telomere length maintenance in bladder and non-small cell lung cancer [36, 37]. None of the other analyzed *TERT* SNVs in our study showed any association with the risk of having AML. Recently, *TERT* 1062A> T SNV (rs35719940) was identified as a mutation with prognostic value and shorter overall survival and it was suggested as an independent negative prognostic factor in AML patients [38]. In our cohort of AML patients, the SNV was identified in 10/226 (4.4%) but it was also found in a similar frequency among Swedish population healthy controls 22/806 (2.7%). This mutations did not significantly alter the risk, nor affecting the OS in our AML cohort, contrary to the study by Salah et al where they identified this mutation in 18 of 153 AML patients and only in one in 197 control group subjects [38]. In the present study we also show that the *TERT* promoter polymorphism rs2853669 influence OS with a significantly shorter survival in AML patients with the CC genotype. In the clinical setting, *NPM1, CEBPA*, and *FLT3-ITD* mutation status is used for risk categorization and prognostic assessment and to guide treatment. Some data suggest that *NPM1* mutant/*FLT3-ITD* wild-type and CEBPA double mutation/FLT3-ITD wild-type AML patients may not benefit from early allogeneic stem cell transplantation, while stem cell transplantations reduce risk of relapse and death for AML patients with *FLT3*-ITD in the absence of *NPM1* or CEBPA double mutation and, thus, represents a preferred treatment option in this high risk group [39]. However, there are still patients without *FLT3-ITD*/*NPM1 or CEBPA* double mutations in which additional prognostic biomarkers would be of high value to further assess the prognosis of the intermediate risk NK-AML patient group. Our results indicate that the *TERT* SNV rs2853669 CC may present an independent biomarker associated with poor prognosis in NK-AML patients.

In this study, we also screened our AML cohort for the two frequent promoter mutations, −228C> T and −250C > T, found in several other types of cancer. Both these mutations were absent in our cohort, confirming the findings by Killela et al and Yan et al on a smaller collection of 15 and 72 AML respectively [5, 18]. However, Yan et al. describe three novel mutations in the N-terminal of TERT (c.896 G > A, Glu280Lys; c.1079 C > G, Leu341Val and c.1451 G > C, Val465Leu) in 72 AML which may lead to shortened telomeres and telomerase dysfunction. AML patients with shorter overhang length had a higher frequency of unfavorable karyotype abnormalities. Therefore, Yan et al deduced that shorter telomere overhang length may indicate poor prognosis for AML patients [18]. Claudia et al showed that modulation of the telomerase complex is a highly effective strategy for targeting leukemia stem cells (LSCc) in AML. They proved that treatment with telomerase inhibitor (imetelstat) prevented the *in vivo* expansion of AML cells, and this was sustained during treatment. Telomerase deficient LSC population, depletes LSCs via the DNA damage, differentiation and apoptosis cascade, suggesting that clinical trials should address the efficacy of telomerase inhibitors as a strategy for preventing relapse or potentially together with chemotherapy, to improve outcomes in AML patients [40].

In addition to TERT, POT 1 is known as a negative regulator of telomere length by directly inhibiting telomerase activity, or by controlling telomeric DNA access to telomerase in human cells [9]. Andrew and collaborators found twelve somatic mutations in *POT1* in 5% of CLL cases, nine among them were detected in N-terminal OB domains for *POT1* and three of the twelve mutations lead to a truncated protein [10]. These somatic mutations were absent in our AML patients. The absence of *TERT* and *POT1* mutations in our cohort indicate that in AML patients, SNVs in *TERT* may be of a relatively high importance for gene expression and/or activity.

Further, *TERT* has been implicated to possess several other cellular functions, such as cell differentiation and apoptosis and most recently as a co-activator to the nuclear transcription factor NFκB, to stimulate the transcription of pro-inflammatory and angiogenic factors [15]. In the present study, we found on a subset of samples where RNA were available, that the CC variant of rs2853669 is associated with an increased expression of IL-6 and TNFα, cytokines considered as markers for inflammation and cancer progression [15]. In the same context Fuxia et al showed that TERT rs2736100 CC enhance the expression of IL-6 in non-small cell lung cancer (NSCLC) in a NFkB dependent way [16]. Inflammation is a beneficial acute response activated to restore tissue injury and as a response to exposure for pathogenic agents. However, if unregulated, it may become chronic, inducing malignant cell transformation in the surrounding tissue [41]. The inflammatory response shares and influences various molecular targets and signaling pathways in carcinogenesis such as apoptosis, increased proliferation rate, and angiogenesis and represent an enabling characteristic that facilitates tumor cell survival [42]. Furthermore, the use of nonsteroidal anti-inflammatory drugs (NSAIDs) has been shown to decrease the amount on several pro-inflammatory mediators as well as incidence and mortality of several cancers [43]. Wang et al observed increased cell apoptosis and demonstrated a strong inhibition of tumor growth, after inhibiting the expression of FLT3 and NFkB p65 simultaneously in THP-1 cell line, and that a combined treatment strategy may be effective for AML in humans [44]. Although, we were not able to demonstrate an increased mRNA expression of *TERT*, in relation to SNV genotypes, an increased IL-6 expression was clearly observed (Supplementary Figure S1) and demonstrates that TERT orchestrates several activities in addition to increased telomere length and cell survival, which contributes to tumor progression.

## MATERIALS AND METHODS

### Study subjects

Two hundred twenty six (*n* = 226) patients mean age 57.7 (range 18–81) with AML from three different Swedish centers with clinical characteristics are shown in Table 5. Blood and bone marrow samples collected at diagnosis before treatment initiation was used for genotyping. Patients diagnosed before 2005 received treatment according to regional guidelines, which most commonly comprised of Cytarabine (AraC) doses of 200 mg/m^2^ as 24 h i.v infusions for 7 days, together with either Daunorubicin or Idarubicin for three days (24). From 2005, patients were treated according to national guidelines (http://www.sfhem.se/Filarkiv/Nationella-riktlinjer, accessed 2013–08-30), where the large majority of cases received induction treatment regimens including Daunorubicin 60 mg/m^2^/day for three days combined with AraC as 1000 mg/m^2^ twice a day in 2 h i.v infusions for 5 days. Other drugs used in combination with AraC and Daunorubicin or Idarubicin included mitoxantrone, 6-Thioguanine, etoposide and cladribine. Survival times were calculated as the time from diagnosis until an event (progression or death) or the latest follow up date. Patients were divided into high, intermediate or low risk group according to standard cytogenetic analysis, or, for NK-AML patients, according to *FLT3*-ITD/*NPM1/CEBPA* mutation status. Thus, *FLT3*-ITD positive/*NPM1* mutation negative NK-AML were considered as high risk, *FLT3*-ITD negative/*NPM1* mutation positive or *FLT3*-ITD negative/biallelic *CEBPA* mutation patients were considered as low risk, and *FLT3*-ITD positive/*NPM1* mutation positive or *FLT3*-ITD negative/*NPM1* mutation negative or *FLT3*-ITD negative/monoallelic and or *CEBPA* wild type patients were considered as intermediate risk. The control population comprised of 806 healthy individuals (50% women, 50% men) with mean age of 54 years (range 20–80 years), randomly collected from the population register from the same geographic region, south-eastern Sweden, as the patients. The study was approved by the local ethical committee and conducted in compliance with the Helsinki declaration.

**Table 5:** AML patient characteristics

AML patient characteristics	Total *N* = 226
***Gender***	
Male	124 (54.86%)
Female	102 (45.13%)
**Age at diagnosis, mean (range)**	57.7 (18–81)
***FLT3* status**	
FLT3 wild type	160 (70.79%)
FLT3 mutated	61 (26.99%)
Not determined	5 (2.21%)
***NPM1* status**	
NPM1 wild type	147 (65.04%)
NPM1 mutated	72 (31.85%)
Not determined	7 (12.04%)
***CEBPA* status**	
Monoallelic	29 (12.83%)
Biallelic	2 (0.88%)
**Karyotype**	
**Normal Karyotype**	169 (74.77%)
NK-low risk	31 (13.71%)
NK-intermediate risk	121 (53.53%)
NK-high risk	17 (7.52%)
**Aberrant Karyotype**:	43 (19.02%)
Aberrant -low risk	5 (2.21%)
Aberrant -intermediate risk	21 (9.29%)
Aberrant -high risk	17 (7.52%)
**Not determined**	14 (6.19%)
**Treatment^a^**	
Dnr + AraC	163 (72.12%)
Ida + AraC	20 (8.85%)
Ida + AraC + CdA	15 (6.63%)
AraC	10 (4.42%)
Dnr + AraC + 6-TG	4 (1.76%)
Dnr + mylotarg	2 (0.88%)
Dnr + AraC + Mitox	2 (0.88%)
Others	10 (4.42%)
**Treatment response^b^**	
CR	167 (73.89%)
Non-CR	32 (11.16%)
Not evaluated	27 (11.94%)

a Dnr = Daunorubicine; AraC = Cytarabine; 6-TG = 6-thioguanine; Ida = Idarubicine; Cda = Cladribine; Mitox = Mitoxantrone; mylotarg = Gemtuzumab ozogamicin

b CR = complete remission

### DNA and RNA extraction

Genomic DNA was isolated from blood samples with the Wizard^®^ Genomic DNA Purification Kit (Promega, Madison, WI) according to the supplier's recommendations. Total RNA was isolated from pelleted cells and vital frozen cells stored at −70°C using RNeasy Mini Kit (Qiagen, Hilden, Germany).

### Mutation analysis

The *TERT* core promoter and 1062A> T polymorphism were amplified using MyTaq^TM^ DNA polymerase (Bioline, USA) and published primers [5, 6], Supplementary Table S1. PCR products were purified with ExoSap-IT (GE Healthcare, USA), and standard Sanger sequencing was performed according to BigDye 3.1 protocol (Applied Biosystems, USA) and capillary electrophoresis on ABI 3500 Genetic Analyzer (Applied Biosystems, USA). PCR and mutation analysis of *POT1* were performed according to the same protocol as for *TERT*, for the exons previously shown to harbor mutations (exons 5–10 and 18) including the oligonucleotide-/oligosaccharide-binding (OB) regions. Primers are listed in Supplementary Table S1.

Insertion mutations in exon 12 of the *NPM1* gene and ITDs in the *FLT3* gene were identified by PCR as previously described [45, 46].

Four overlapping primer pairs were used to amplify the entire CEBPA coding region, with subsequent DNA sequencing according to the Big Dye 3.1 protocol (Supplementary Table S1).

### *TERT* SNVs and genotyping

For this study we selected 4 tag *TERT* SNVs described to be associated with risk in other tumors [8, 47–50]. The rs2853669, rs2736100 and rs4246742 in *TERT*, were genotyped using TaqMan^®^ SNV Genotyping assays (C_8773290_10, C_1844009_10, C_11772271_20, respectively). All analysis were performed in ABI Prism 7500 or 7900 Sequence Detection System (Applied Biosystems), using the SDS 1.3 and 2.4 software for allelic discrimination, respectively.

The rs10069690 and *TERT* 1062A > T (rs35719940) were genotyped in AML patients and healthy controls by pyrosequencing. Each PCR reaction consisted of 5X My Taq Reaction Buffer, 1 U My Taq DNA polymerase (Bioline, USA), 1 μM of each primer (Supplementary Table S1), and 20 ng of template DNA in a final volume of 20 μl.

### cDNA synthesis and quantitative real-time PCR

Thirty six samples were available for RNA isolation and investigated for *TERT, IL-6, IL-1β* and *TNFα* gene expression in relation to the rs2853669 genotype. Twelve samples were (TT), eleven (TC) and thirteen (CC). Total RNA from each sample was reversely transcribed into cDNA with MaximaR First Strand cDNA synthesis Kit for RT-qPCR (Fermentas, St Leon-Rot, Germany) according to supplier's instructions. The relative mRNA expression of *TERT*, *IL-6, IL-1β* and *TNFα* was determined by real-time PCR [7900HT Fast Real-Time PCR System (Applied Biosystems)], and normalized to the expression of two control genes *β-glucuronidase* (*GUSB*) (4333767F, amplicon length 81 bp) and *Hypoxanthin-guanine phosphoribosyltransferase 1* (*HPRT1*) (4333768F, amplicon length 100 bp) (Applied Biosystems). All samples were run as duplicates. The calculation of the normalized gene expression was based on established methods, using the 2^−ΔCT^ formula to calculate final relative expression in relation to *HPRT1* and *GUSB* mRNA expression as house-keeping genes [51].

### Statistical analysis

The genotype distribution in the control population was tested for Hardy-Weinberg equilibrium using a χ2 test and all SNV's displayed an expected Hardy-Weinberg distribution. The association between each SNVs and risk of AML are presented as odds ratios (OR) with 95% confidence intervals (CI). For survival analysis Kaplan-Meier, curves were generated and tested for significance by the log-rank test. Overall survival was the time elapsed from diagnosis of cancer to the date of death or date of the latest follow up. These tests were performed with both the entire material and in the NK-AML subset. Significant findings were also further investigated by multivariable Cox regression analysis using the forced entry method. Statistical analysis was performed with IBM SPSS Statistics software version 20 (IBM Corporation, Somers, NY) and Epi info™7. A *p*-value of < 0.05 was considered as significant.

## CONCLUSIONS

In conclusion, our results show an association of the *TERT* SNV rs2853669 with the risk of having AML, a possible co-regulation of cytokine expression, as well as an association between *TERT* SNV genotype and OS. Our results indicate that the SNV rs2853669 has potential as a prognostic marker of survival in AML, in addition to the clinically used biomarkers *FLT3-ITD, NPM1* and *CEBPA* mutations, which may further aid in treatment decisions such as allocation of patients to early stem cell transplantation.

## SUPPLEMENTARY METHODS FIGURE AND TABLE


