# Supplementary material for: Association between TERT promoter polymorphisms and acute myeloid leukemia risk and prognosis
Source: Oncotarget. 2015 Jul 23;6(28):25109–20. doi: 10.18632/oncotarget.4668 (PMC4694818; doi:10.18632/oncotarget.4668)
Supplement: Supplementary file 1 [file oncotarget-06-25109-s001.pdf]

SUPPLEMENTARY METHODS

Mutations analysis

TERT core promoter, *TERT* 1062 A > T and *POT1*

PCR was performed in a total volume of 20 µl, containing 5X MyTaq Reaction Buffer (comprising 1 mM dNTP and 3 mM $MgCl_2$), 1 µM for each pair primers (Supplementary Table S1), 1 U MyTaq™ DNA polymerase and 20 ng of template DNA. After an initial heat activation step at 95°C for 2 minutes, thirty five cycles of PCR were performed with the following cycling conditions: 95°C for 30 s, 61°C for 30 s, and 72°C for 30 s.

CEBPA mutations

The total reaction volume of 20 µl contained 20 ng of DNA, 1 µM of each primer, 1 U MyTaq™ DNA polymerase, 5X MyTaq Reaction Buffer, and 1 µl of 100% dimethyl sulfoxide. The following PCR conditions were used: incubation at 94°C for 3 min, 35 cycles at 94°C for 45s (denaturation), 63°C for 45s (annealing) and 72°C for 1 min (extension) followed by the final extension at 72°C for 10 min. PCR products were sequenced in both directions forward and reverse for each primer using the ABI 3500 Genetic Analyzer (Applied Biosystems, USA). NM_004364.4 CEBPA ID transcript was used to align the derived sequences.

PCR TaqMan® SNP genotyping Genotyping

A Total volume of 10 µl per well contained 5 µl TaqMan® Universal PCR Master Mix (2X), 0.25 µl assay mix (40X), including the two allele-specific TaqMan® MGB probes containing distinct fluorescent dyes (FAM and VIC) and a PCR primer pair to detect specific SNV targets and 20 ng of DNA.

Pyrosequencing

The cycling conditions were 95°C for 2.5 min, followed by 35 cycles of 95°C denaturation for 30 s, 66.1°C and 58°C for annealing for rs10069690 and A1062T (rs35719940) mutation, respectively, and 30 s extension at 72°C for 30 s and with a final extension at 72°C for 5 min. Successful PCR was determined by 1.5% agarose gel electrophoresis and the genotypes were assessed on a PSQ96MD instrument (Qiagen, Sweden) as previously described [1]. In brief, the amplified and biotinylated PCR product was isolated with a Vacuum Prep Workstation (Qiagen, Sweden). The sequencing primers (Invitrogen, Paisley, UK) 5′-gggtgaggtggacaga-3′ and 5′-tgggggccaagggcg-3′for rs10069690 and rs35719940 respectively was annealed to the single-stranded DNA template for 2 min at 80°C. The plate was then transferred to the PSQ96MD instrument and sequencing was performed with the following dNTP dispensing order: cgt agc tgc and tcg atc gct for the polymorphism and the mutation respectively.

Quantitative real-time PCR

The relative mRNA expression was determined by real-time PCR with the TaqMan® Fast Universal PCR Master Mix (Applied Biosystems) and cDNA-specific primer/probe mixes for *TERT*, *IL-6, IL-1β* and *TNFα* (Hs00972656_m1, amplicon length 79 bp, Hs00985639_m1, amplicon length 66 bp, Hs01555410_m1, amplicon length 91 bp and Hs01113624_g1, amplicon length 143 bp, respectively) (Applied Biosystems). The qPCR mix contained 2 µl of sample cDNA, 5 µl of TaqMan Fast Universal Master mix (2X), 0.5 µl assay mix and 2.5 µl of mili-Q water with the following cycling conditions; 20 s in 95°C and 40 cycles consisted of 3 s in 95°C, 30 s in 60°C.

REFERENCE

1. Green H, Soderkvist P, Rosenberg P, et al. mdr-1 single nucleotide polymorphisms in ovarian cancer tissue: G2677T/A correlates with response to paclitaxel chemotherapy. Clin Cancer Res. 2006; 12:854–859.

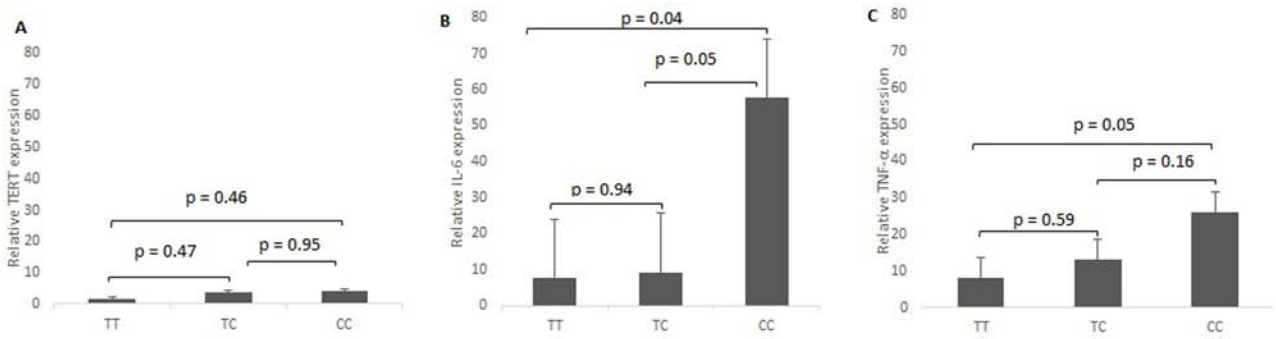

Supplementary Figure S1: Expression levels of TERT, IL-6 and TNFα in different rs2853669 genotypes, TERT is 2.17 fold enhanced in CC genotype compared to TT (*p* = 0.46) A. however IL-6 is 6.25 fold enhanced in CC genotype compared to TT (p = 0.04) B. and TNFα expression was 3.58 fold higher with CC genotype (p = 0.05) C.

Supplementary Table S1: *TERT*, *POT1* and *CEBPA* primers

	Forward primer	Reverse primer
TRET promoter	GGCCGATTCGACCTCTCT	AGCACCTCGCGGTAGTGG
TERT EX15	AGCTTTCCGGTGTCTCCTG	TCTGCACTTCAGCAGCATCT
TERT rs10069690	biotin-TCCTGGCCGCATGTGTGT	AGAGTGTGGGGTGAGGTGGA
TERT rs35719940	TCTCCTGGGAGGGGAGCT	biotin-AGGAGTGGCACGTAGGTGACA
POT1-EX5	AGCATGTAATCACATTGGAGGT	TCTATGTGTGTGGCATATACAGG
POT1-EX6	CATGGATTTGCTGCTAATATGAT	TGGAACCGTGTTCCTAAATC
POT1-EX7	TGAAAGCCAAGAAAATGTCTC	TGAAAAGCTTGCTGTCATGT
POT1-EX8	TTCCAGTTTCTTTGGTTCGT	CACAGCATGCTTTATCTCATCA
POT1-EX9	TGCCAATATTCAGAGGCATAAG	AAAAATTTACATGAGCAAAAATCAC
POT1-EX10	ATTGGCCCCATATTGGTTAT	GCACAAAAGGCTAGGGAACT
POT1-EX18	TCTAGATTATCTTTTAATTGGACA	ACTTCTAAAAGTCCACAGAGTACA
CEBPA-1	CCTGCCGGGTATAAAAGCTG	AGCCTGCCGTCCAGGTAG
CEBPA-2	GCTGGGCGGCATCTGCGA	CCCCGACGCGCTCGTACAGG
CEBPA-3	CCGGCTACCTGGACGGCAGG	CGTTGCTGTTCTTGTCCACCGACTTCTT
CEBPA-4	GTGGACAAGAACAGCAACGA	AGGCACCGGAATCTCCTAGT
